# Successful Implementation of a Nurse-Led Daily Spontaneous Awakening Trial Within a Modified Extubation Protocol: A Before-and-After Study

**DOI:** 10.7759/cureus.108008

**Published:** 2026-04-30

**Authors:** Kenichi Nitta, Hiroshi Imamura, Hiroshi Kamijo, Sari Shimizu, Momoko Uchida, Mayo Akita, Takuya Kishida, Katsunori Mochizuki, Yuichiro Kashima, Chiaki Toida

**Affiliations:** 1 Advanced Emergency and Critical Care Center, Shinshu University Hospital, Matsumoto, JPN

**Keywords:** extubation, mechanical ventilation, nurse-led daily spontaneous awakening trial, spontaneous awakening trial, weaning protocol

## Abstract

Background

Successful liberation from mechanical ventilation (MV) requires a systematic assessment of readiness for extubation. The 2015 Japanese Society of Intensive Care Medicine guidelines recommend daily spontaneous awakening trials (SATs) paired with spontaneous breathing trials (SBTs) and standardized risk stratification for post-extubation failure. We implemented a modified extubation protocol incorporating a nurse-led daily SAT and an enhanced risk assessment framework. This study aimed to evaluate the feasibility and safety of this implementation compared with the existing protocol (EP).

Methods

In this retrospective before-and-after study, we compared outcomes in patients managed with the EP (March 2017 to February 2020) and those managed with the modified protocol (MP) (March 2021 to February 2024) at the Advanced Emergency and Critical Care Center of Shinshu University Hospital, Matsumoto, Japan. We included adult patients who received MV for ≥48 hours and underwent protocolized extubation. The primary outcomes were the duration of MV, re-intubation rate, and post-extubation respiratory failure (PERF). Statistical analysis included inverse probability of treatment weighting (IPTW) to adjust for baseline differences.

Results

We analyzed 158 and 122 patients in the EP and MP groups, respectively. After IPTW adjustment, there were no significant differences in the duration of MV (median 6.0 vs. 6.0 days, p = 1.0), re-intubation rate (3.8% vs. 3.5%, p = 0.94), or PERF rate (12.7% vs. 10.1%, p = 0.54). The MP group had a significantly higher rate of prophylactic high-flow nasal cannula (HFNC) use (6.2% vs. 21.0%, p < 0.001).

Conclusions

In this exploratory study, the MP did not show statistically significant differences in re-intubation rates or PERF compared to the EP. However, the MP, incorporating nurse-led daily SATs and systematic risk stratification, was associated with a significantly higher use of prophylactic HFNC. The study was underpowered to confirm equivalence, and these findings should be interpreted as hypothesis-generating.

## Introduction

Liberation from mechanical ventilation (MV) is a pivotal step in intensive care. Delayed extubation increases the risk of ventilator-associated pneumonia, intensive care unit (ICU) delirium, and healthcare costs, while premature extubation can lead to respiratory failure, re-intubation, and increased mortality [1-3]. Standardized, protocol-based approaches have been shown to reduce the duration of MV and improve patient outcomes [4,5].

In 2007, our institution implemented an extubation protocol to standardize weaning [6]. Although physician-directed spontaneous awakening trials (SATs) were performed immediately before extubation, they were not formally incorporated into the existing protocol (EP). In February 2015, the Japanese Society of Intensive Care Medicine, in collaboration with the Japanese Respiratory Society and the Japanese Society of Respiratory Care Medicine, published evidence-based guidelines for ventilator discontinuation [7]. These guidelines recommend pairing SATs with spontaneous breathing trials (SBTs), comprehensive risk assessment for post-extubation failure, and preventive strategies for high-risk patients. Based on these recommendations, we revised the EP in 2020 to incorporate nurse-led daily SATs, systematic evaluation of risk factors for post-extubation airway obstruction and respiratory failure, as well as standardized preventive interventions, including prophylactic non-invasive positive pressure ventilation (NPPV), high-flow nasal cannula (HFNC) use, and corticosteroid therapy. This study aimed to evaluate whether the modified protocol (MP) maintained safety while improving liberation from MV.

## Materials and methods

Study design and setting

We conducted this retrospective before-and-after cohort study at the Advanced Emergency and Critical Care Center of Shinshu University Hospital, a 20-bed tertiary care facility in Matsumoto, Japan. The study was approved by the Shinshu University Institutional Review Board, and the requirement for informed consent was waived because of its retrospective nature (Approval Number: 5940).

Study population

We included adult patients (≥18 years) admitted to our center who required MV for ≥48 hours and underwent protocolized extubation. Patients were excluded if they died during MV, underwent tracheostomy, had self-extubation before or after fulfilling the conditions for SBTs, were transferred to our center while receiving MV, or had do-not-resuscitate status. Patients who died during MV were excluded because they never reached the stage of extubation readiness assessment and were therefore never exposed to the extubation protocol under evaluation. This exclusion criterion is consistent with the methodology used in previous extubation and weaning protocol studies [4-6].

Study periods

We compared patient outcomes across two distinct periods. The first cohort included patients treated under the EP from March 1, 2017, to February 29, 2020, spanning three years. The second cohort included patients managed with the MP from March 1, 2021, to February 29, 2024, covering three years. To ensure a clear distinction between the two protocols and avoid confounding, we excluded data from the transitional period between March 2020 and February 2021, during which the MP was gradually introduced and medical staff underwent training on the revised procedures. Notably, COVID-19 patients at our institution were managed in a separate dedicated ICU within the hospital and were not admitted to the Advanced Emergency and Critical Care Center, where this study was conducted.

Protocols

Existing Protocol (EP) (2017-2020)

**Figure 1:**
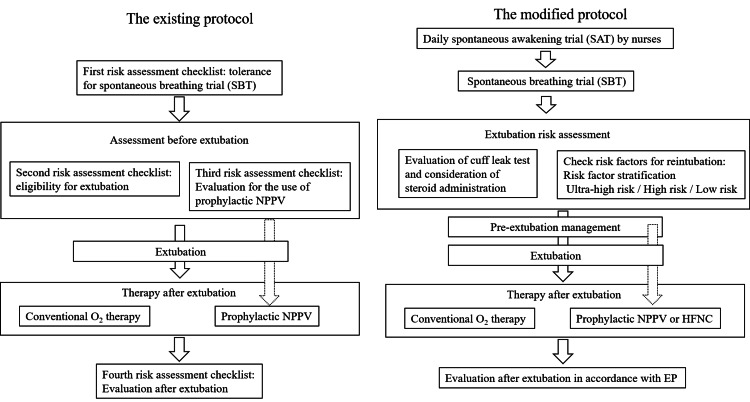
Existing and modified extubation protocols The existing protocol (left) was adapted from Nitta et al. [6], published under the Creative Commons Attribution 4.0 International License (CC BY 4.0). NPPV: non-invasive positive pressure ventilation; HFNC: high-flow nasal cannula; EP: existing protocol

The EP did not incorporate SATs and comprised four sequential risk assessment checklists (Figure 1) [6]. First, tolerance was assessed using SBTs. If patients passed, the second and third checklists were evaluated simultaneously.

In the second stage, patients were required to meet all seven criteria for extubation eligibility. If eligible, extubation was performed; otherwise, MV was continued with daily reassessment. In the third stage, if a patient met at least one of the three criteria for post-extubation risk, the use of prophylactic NPPV was considered. The final decision on the use of prophylactic NPPV was left to the discretion of the attending physicians. The fourth stage involved post-extubation evaluation within 48 hours. Attending physicians performed assessments 60 minutes after extubation and during morning and evening rounds. In addition, ICU nurses conducted hourly physical assessments, alerting physicians to any abnormalities among the six specified criteria. Rescue NPPV was applied following the protocol proposed by Kikuchi et al. [8], which comprised six checklists: (1) the need for ventilatory assistance, (2) eligibility for NPPV, (3) effectiveness evaluation at 30-120 minutes after the start of NPPV, (4) effectiveness evaluation at 12-24 hours after the start of NPPV, (5) eligibility for weaning, and (6) evaluation at 30-120 minutes after discontinuation of NPPV. We used the first four of these checklists, omitting the fifth and sixth. For patients who did not fulfill each checklist, re-intubation was performed.

Modified Protocol (MP) (2021-2024)

**Figure 2:**
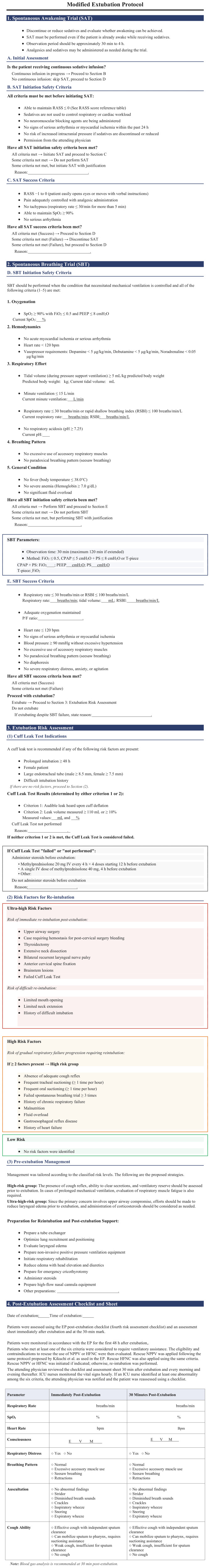
Details of the modified extubation protocol RASS, Richmond Agitation-Sedation Scale; FiO_2_, fraction of inspiratory oxygen; SpO_2_, arterial oxygen saturation; PEEP, positive end-expiratory pressure; CPAP, continuous positive airway pressure; PS, pressure support; IV, intravenous injection; RR, respiratory rate; RSBI, rapid shallow breathing index; NPPV, non-invasive positive pressure ventilation; HFNC, high-flow nasal cannula; EP, existing protocol

The MP included several additions and modifications to the EP (Figure 1). Figure 2 presents the details of the MP. In the EP, physicians performed SAT at their discretion and without explicit criteria. In the MP, nurse-led SATs were conducted daily for all MV patients receiving continuous sedation, along with daily screening for SBT eligibility and subsequent SBT implementation. A SAT is a planned, temporary interruption or reduction of continuous sedation to assess a patient’s neurological status, level of consciousness, and ability to protect the airway. The purpose of daily SATs is to minimize unnecessary sedation, thereby facilitating earlier recognition of extubation readiness and reducing complications associated with prolonged sedation, such as ICU delirium and ventilator-associated pneumonia. As part of the implementation of the ICU Liberation bundle, adherence to nurse-led SAT was reviewed during daily afternoon multidisciplinary conferences [9]. The MP included comprehensive risk assessment measures, such as cuff leak tests (CLTs) for patients at risk of post-extubation airway obstruction, evaluation of risk factors for re-intubation, and standardized preventive interventions based on risk stratification.

Risk assessment identified specific high-risk features for post-extubation airway obstruction, including intubation duration ≥48 hours, female sex, a large endotracheal tube (defined as 8.5 mm or larger for males and 7.5 mm or larger for females), and a history of difficult intubation. For re-intubation risk, patients were stratified into high- and ultra-high-risk categories. High risk was defined as the presence of two or more of the following factors: inadequate cough, frequent suctioning requirements of more than once per hour, three or more SBT failures, chronic respiratory failure, malnutrition, or fluid overload. Ultra-high-risk patients included those who had undergone upper airway surgery, thyroidectomy, extensive neck dissection, bilateral recurrent laryngeal nerve palsy, anterior cervical spine fixation, brainstem lesions, or failed CLTs.

Preventive interventions were tailored according to risk assessment and included prophylactic corticosteroids for patients with failed CLTs, prophylactic NPPV or HFNC for high-risk patients, and positioning strategies and pulmonary hygiene measures to optimize post-extubation outcomes. Prophylactic NPPV or HFNC was initiated immediately after extubation in patients identified as high-risk through the risk stratification framework, before any signs of respiratory failure developed. Prophylactic NPPV or HFNC was indicated for high-risk patients (≥2 risk factors for re-intubation) and ultra-high-risk patients. The decision to use prophylactic NPPV or HFNC was left to the discretion of the attending physician.

Following extubation, patients were assessed using the EP post-extubation checklist (fourth risk assessment checklist) and an assessment sheet immediately post-extubation and at the 30-minute mark. Patients were monitored in accordance with the EP for the first 48 hours after extubation. The attending physician reviewed the checklist and assessment sheet 30 minutes after extubation and every morning and evening thereafter. ICU nurses monitored vital signs hourly. If an ICU nurse identified at least one abnormality among the six criteria, the attending physician was notified, and the patient was reassessed using the checklist.

Patients who met at least one of the six criteria were considered to require ventilatory assistance. Rescue NPPV was applied following the same protocol proposed by Kikuchi et al. [8], as used in the EP. In the MP, rescue HFNC was also incorporated as an additional option and was applied using the same criteria. Eligibility for rescue NPPV or HFNC, including contraindications, was then evaluated. The decision to use rescue NPPV or HFNC was left to the discretion of the attending physician. Rescue NPPV or HFNC was initiated if indicated; otherwise, re-intubation was performed.

Data collection

Data were extracted from patients’ medical records, including demographics, comorbidities (hypertension, diabetes mellitus, chronic obstructive pulmonary disease (COPD), coronary artery disease, congestive heart failure, and chronic renal failure), Acute Physiology and Chronic Health Evaluation (APACHE) II score [10] at ICU admission, and clinical outcomes.

Outcomes

The primary outcomes were duration of MV, re-intubation within 48 hours, and post-extubation respiratory failure (PERF). PERF was defined as re-intubation or the requirement for rescue NPPV or HFNC within 48 hours. Failure to wean from prophylactic NPPV or HFNC within 48 hours of extubation was also classified as PERF. The same six post-extubation monitoring criteria were used to identify the need for ventilatory assistance in both study periods, ensuring consistent assessment of PERF. The criteria for initiating rescue NPPV or HFNC were standardized and did not change between the EP and MP periods.

Secondary outcomes included critical care center (CCC) length of stay (LOS), hospital LOS, 28- and 60-day mortality, hospital mortality, use of prophylactic NPPV or HFNC, and corticosteroid use for failed CLTs. In addition, a cause-of-death analysis was performed for all hospital mortality cases to determine whether deaths were related to the extubation protocol. The cause of death, the relationship to airway or respiratory complications following extubation, and the time from extubation to death were evaluated.

Statistical analysis

Continuous variables were presented as means ± standard deviation or median (interquartile range, IQR), as appropriate. Categorical variables were presented as numbers (percentages). Between-group comparisons were performed using the Mann-Whitney U test for continuous variables and the chi-square test or Fisher’s exact test for categorical variables. To account for potential confounders, we performed inverse probability of treatment weighting (IPTW) using propensity scores estimated using a logistic regression model. The propensity score model included age, sex, comorbidities, and APACHE II score. After IPTW adjustment, we assessed covariate balance using standardized mean differences, with values <0.10 considered acceptable.

Continuous outcomes were compared using IPTW-adjusted analysis. Because all continuous variables were non-normally distributed, weighted medians and IQRs were reported. Differences in weighted medians with 95% confidence intervals (CIs) were estimated using 2,000 bootstrap resamples. Weighted mean differences with robust standard errors were also calculated using survey-weighted linear regression. Binary outcomes were compared using IPTW-adjusted analysis. Risk differences were estimated using weighted linear regression with robust standard errors. Risk ratios (RRs) with 95% CIs were estimated using 2,000 bootstrap resamples. Weighted odds ratios (ORs) were estimated using logistic regression as a sensitivity measure.

All analyses were performed using R v.4.5.2 (R Foundation for Statistical Computing, Vienna, Austria) with EZR software [11]. Statistical significance was defined as a two-sided p-value <0.05.

## Results

Patient characteristics

**Figure 3:**
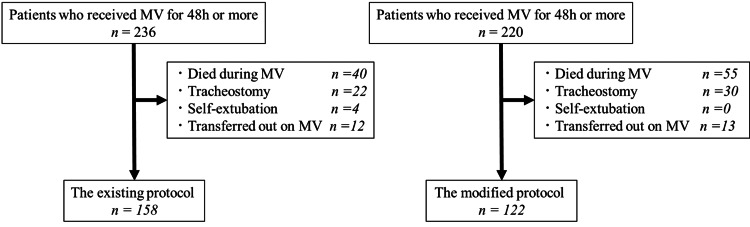
Flow chart of the study patients for the existing and the modified extubation protocol MV: mechanical ventilation

During the study period, 280 patients met the inclusion criteria: 158 in the EP group and 122 in the MP group (Figure 3). As shown in Figure 3, self-extubation occurred in four patients in the EP group and in no patients in the MP group. The four self-extubated patients in the EP group were excluded from the primary analysis because they did not undergo protocolized extubation. Among these four patients, three required re-intubation without delay following self-extubation, while one was managed successfully without re-intubation. No tracheostomies were performed. All four patients subsequently underwent protocolized extubation, and none developed PERF. Three patients survived to hospital discharge; one patient died on hospital day 113 due to progression of the underlying disease, unrelated to the self-extubation event. During the EP period, 40 of 236 patients (16.9%) died during MV before reaching extubation readiness assessment, compared with 55 of 220 patients (25.0%) during the MP period. In the EP group, protocol violations occurred in two patients (1.3%), who were extubated without adherence to the EP. Protocol violations were not observed in the MP group. Patients who violated the protocol were not excluded from the analysis. The baseline characteristics are presented in Table 1.

**Table 1:** Baseline characteristics Baseline characteristics of patients in the existing protocol (EP) and modified protocol (MP) groups before inverse probability of treatment weighting. Continuous variables are presented as medians (interquartile ranges), and categorical variables are presented as n (%). p-values were calculated using the Mann-Whitney U test (W) for continuous variables and the chi-square test (χ²) for categorical variables. Fisher’s exact test was used when any expected cell count was <5; no test statistic is reported for these variables. SMD, standardized mean difference; COPD, chronic obstructive pulmonary disease; CAD, coronary artery disease; CHF, congestive heart failure; CRF, chronic renal failure; APACHE II, Acute Physiology and Chronic Health Evaluation II

Variable	EP group (n = 158)	MP group (n = 122)	Test statistic	p-value	SMD
Age (years)	71 (61-79)	71 (62-78)	W = 9942.5	0.651	0.054
Male, n (%)	113 (71.5)	78 (63.9)	χ² = 1.49	0.222	0.116
Hypertension, n (%)	73 (46.2)	49 (40.2)	χ² = 0.79	0.374	0.122
Diabetes mellitus, n (%)	28 (17.7)	31 (25.4)	χ² = 2.01	0.157	0.188
COPD, n (%)	5 (3.2)	7 (5.7)	-	0.376	0.125
CAD, n (%)	12 (7.6)	20 (16.4)	χ² = 4.43	0.035	0.273
CHF, n (%)	20 (12.7)	16 (13.1)	χ² < 0.01	1.000	0.014
CRF, n (%)	17 (10.8)	8 (6.6)	-	0.291	0.150
APACHE II score	15 (10-21)	17.5 (11-22)	W = 8797.5	0.211	0.116

After IPTW adjustment, all standardized mean differences were <0.10, indicating good balance between the groups (Table 2).

**Table 2:** Baseline characteristics after IPTW Baseline characteristics of patients in the existing protocol (EP) and modified protocol (MP) groups after inverse probability of treatment weighting (IPTW). Continuous variables are presented as weighted median (interquartile range) and categorical variables as weighted n (%). p-values were calculated using IPTW-adjusted comparisons. All standardized mean differences (SMDs) were <0.10, indicating adequate covariate balance between the groups. COPD, chronic obstructive pulmonary disease; CAD, coronary artery disease; CHF, congestive heart failure; CRF, chronic renal failure; APACHE II, Acute Physiology and Chronic Health Evaluation II

Variable	EP group (n = 157.6)	MP group (n = 123.0)	p-value	SMD
Age (years)	70.7 (60-79)	71.4 (62-79)	0.950	0.021
Male, n (%)	107.7 (68.4)	84.6 (68.8)	0.946	0.009
Hypertension, n (%)	70.6 (44.8)	55.6 (45.2)	0.946	0.009
Diabetes mellitus, n (%)	31.5 (20.0)	24.4 (19.8)	0.972	0.004
COPD, n (%)	6.7 (4.2)	5.3 (4.3)	0.971	0.004
CAD, n (%)	18.7 (11.9)	14.3 (11.6)	0.950	0.008
CHF, n (%)	18.4 (11.7)	13.9 (11.3)	0.928	0.011
CRF, n (%)	13.8 (8.8)	11.6 (9.4)	0.879	0.023
APACHE II score	16 (10-22)	16 (10.5-22)	0.811	0.005

Primary outcomes

There were no significant differences in the duration of MV, rate of re-intubation, or PERF between the two groups before or after IPTW adjustment (Table 3).

**Table 3:** IPTW-adjusted comparison of continuous outcomes Values are presented as weighted medians (interquartile ranges). The differences and 95% confidence intervals (CIs) were estimated using 2,000 bootstrap resamples. IPTW, inverse probability of treatment weighting; MV, mechanical ventilation; CCC, critical care center; LOS, length of stay; MP, modified protocol; EP, existing protocol

Variable	EP group	MP group	Difference (95% CI)	p-value
Primary outcome				
MV duration, days	6 (4, 10)	6 (4, 11)	0 (-2 to 1)	1.000
Secondary outcomes				
CCC days, days	20 (10, 35)	16 (9, 34)	-4 (-8 to 2)	0.246
Hospital LOS, days	36 (25, 55)	32 (23, 50)	-4 (-10 to 2)	0.214

Secondary outcomes

The MP group exhibited a significantly higher use of prophylactic HFNC (6.2% vs. 21.0%, p < 0.001, after IPTW) (Table 4). Table 5 presents a detailed breakdown of PERF in the EP and MP groups. There were no significant differences in prophylactic NPPV use, failed CLTs, steroid use, CCC LOS, hospital LOS, hospital mortality, 28-day mortality, or 60-day mortality between the two groups, both before and after IPTW adjustment (Tables 3-4). A cause-of-death analysis revealed that all hospital deaths in both groups were unrelated to the extubation protocol (Table 6). No deaths were attributable to airway or respiratory complications following extubation, and no deaths occurred within 48 hours of extubation. The median time from extubation to death was 24.5 days in both groups (EP: IQR 9-47 days; MP: IQR 20-55 days). The most common cause of death in the MP group was progression of underlying disease (60%), followed by nosocomial infection/sepsis (20%) and withdrawal of life-sustaining treatment (20%).

**Table 4:** IPTW-adjusted comparison of binary outcomes Values are presented as n (weighted %). Risk differences (RDs) and 95% confidence intervals (CIs) were estimated using an IPTW-adjusted linear regression with robust standard errors. The risk ratios (RRs) and 95% CIs were estimated using 2,000 bootstrap resamples. IPTW, inverse probability of treatment weighting; CI, confidence interval; CLT, cuff leak test; HFNC, high-flow nasal cannula; NPPV, non-invasive positive-pressure ventilation

Outcome	EP group	MP group	Effect estimate (95% CI)	p-value
Primary outcomes				
Re-intubation	6 (3.8)	4 (3.5)	RR: 1.00 (0.96 to 1.05)	0.940
			RD: 0.2% (-4.2 to 4.6%)	0.924
PERF	20 (12.7)	12 (10.1)	RR: 0.80 (0.37 to 1.62)	0.540
			RD: -2.6% (-10.3 to 5.1%)	0.504
Secondary outcomes				
Hospital mortality	8 (4.8)	10 (8.6)	RR: 1.80 (0.66 to 5.31)	0.244
			RD: 3.8% (-2.6 to 10.2%)	0.245
28-day mortality	3 (1.9)	2 (1.6)	RR: 0.88 (0.00 to 4.33)	0.814
			RD: -0.2% (-3.3 to 2.9%)	0.882
60-day mortality	6 (3.6)	5 (4.3)	RR: 1.20 (0.36 to 3.97)	0.735
			RD: 0.7% (-3.8 to 5.2%)	0.751
Prophylactic NPPV	15 (9.5)	13 (11.0)	RR: 1.15 (0.52 to 2.59)	0.692
			RD: 1.4% (-6.1 to 9.0%)	0.707
Prophylactic HFNC	10 (6.2)	26 (21.0)	RR: 3.40 (1.72 to 8.69)	<0.001
			RD: 14.8% (6.2 to 23.4%)	0.001
Failed CLT	10 (6.6)	14 (11.2)	RR: 1.70 (0.59 to 4.72)	0.305
			RD: 4.6% (-4.1 to 13.3%)	0.302
Steroids use	13 (8.1)	14 (11.2)	RR: 1.37 (0.52 to 3.56)	0.473
			RD: 3.0% (-5.6 to 11.7%)	0.494

**Table 5:** Breakdown of post-extubation respiratory failure using respiratory support modality in the existing and modified protocol groups PERF, post-extubation respiratory failure; NPPV, non-invasive positive-pressure ventilation; HFNC, high-flow nasal cannula; NIV, non-invasive ventilation

MP group	Total	PERF, n	Post-NIV re-intubation, n
Prophylactic NPPV, n	16	1	1
Prophylactic HFNC, n	27	4	0
Rescue NPPV, n	7	7	1
Rescue HFNC, n	1	1	1
Re-intubation, n	2	2	-
EP group			
Prophylactic NPPV, n	15	1	0
Prophylactic HFNC, n	9	2	0
Rescue NPPV, n	12	12	0
Rescue HFNC, n	4	4	0
Re-intubation, n	6	6	-

**Table 6:** Cause-of-death analysis for hospital mortality cases Data are presented as n (%) or median (interquartile range). EP, existing protocol; MP, modified protocol; IQR, interquartile range

	EP group (n = 8)	MP group (n = 10)
Cause of death		
Progression of underlying disease, n (%)	2 (25)	6 (60)
Nosocomial infection/sepsis, n (%)	3 (37.5)	2 (20)
Cardiovascular event, n (%)	0 (0)	0 (0)
Respiratory failure (post-extubation), n (%)	0 (0)	0 (0)
Withdrawal of life-sustaining treatment, n (%)	3 (37.5)	2 (20)
Other, n (%)	0 (0)	0 (0)
Relationship to extubation protocol		
Airway/respiratory-related death, n (%)	0 (0)	0 (0)
Death unrelated to extubation protocol, n (%)	8 (100)	10 (100)
Timing and details		
Time from extubation to death, days, median (IQR)	24.5 (9-47)	24.5 (20-55)
Death within 48 hours of extubation, n (%)	0 (0)	0 (0)

## Discussion

In this study, we evaluated the safety and efficacy of the MP for ventilator weaning and extubation in accordance with the 2015 Japanese Society of Intensive Care Medicine guidelines. The primary outcomes - duration of MV, re-intubation rate, and PERF - showed no significant differences between the EP and MP groups. Implementation of a nurse-led daily SAT protocol with enhanced risk assessment maintained safety without prolonging the duration of MV.

Nurse-led daily SATs and duration of MV

After IPTW adjustment, the median duration of MV was six days for both groups (p = 1.000). The literature suggests that SAT reduces oversedation and enables an appropriate assessment of patients' consciousness levels and respiratory muscle strength [5,12]. Nurse-driven SAT protocols achieve similar or better patient outcomes than physician-led approaches, with no increase in adverse events [13,14]. In our study, the MP incorporated SAT as a mandatory component with an observation period of approximately 30 minutes to 4 hours. Although the duration of MV was not reduced, outcomes were comparable between protocols. Complications associated with SATs include self-extubation. In randomized trials, self-extubation rates were slightly higher with protocolized SATs; however, re-intubation rates and overall safety profiles were equivalent [5,15], and nurse-driven protocols have not been associated with increased short-term adverse effects or mortality [13,14]. Staff concerns regarding workload and adverse events are common but can be mitigated through education, protocol standardization, and a supportive unit culture [16,17]. In this study, nurse-led SAT was implemented safely over a three-year period, with no cases of self-extubation during SAT.

An important advantage of the MP is the standardization of the assessment process and the redistribution of daily screening responsibilities from physicians to nursing staff. Under the EP, SATs were performed at the physician's discretion without explicit criteria; the MP ensures that all patients receive consistent, evidence-based evaluation regardless of which physician is on duty. In our ICU setting, this ensures daily assessment even during periods of high physician workload and reduces the risk of delayed recognition of extubation readiness. Notably, self-extubation events leading to study exclusion occurred in four patients in the EP group and none in the MP group. Although these patients were excluded from the primary analysis, precluding direct comparison, the absence of self-extubation in the MP group, despite the introduction of daily SAT, suggests that the structured SAT protocol with standardized monitoring did not increase the risk of unplanned extubation in our cohort. The four self-extubated patients in the EP group were excluded from the primary analysis because they did not undergo protocolized extubation. These outcomes suggest that the exclusion of self-extubated patients did not materially affect the study conclusions.

PERF and Re-intubation

The incidence of PERF decreased from 12.7% in the EP group to 10.1% in the MP group; however, the difference was not statistically significant (IPTW-adjusted, p = 0.540). The point estimate for PERF suggested a potential relative risk reduction (RR: 0.80, 95% CI: 0.37-1.62); however, the wide CI precludes definitive conclusions, and the lack of statistical significance may reflect insufficient sample size. The systematic risk assessment incorporated into the MP may have facilitated a more accurate assessment of extubation readiness. However, whether this translates into a clinically meaningful reduction in PERF remains unclear. Importantly, the absence of increased re-intubation rates or PERF in the MP group suggests that the protocol modifications did not compromise patient safety. A larger multicenter study is needed to more precisely estimate the effects of the MP on clinical outcomes.

Previous studies have demonstrated that protocolized ventilator weaning strategies can reduce the duration of MV and decrease re-intubation rates [4-6]. The re-intubation rates in this study were already very low in both groups (3.5%-3.8%), suggesting a floor effect that limits the ability to demonstrate further reduction. In contrast, the PERF rate of 10%-13% does not represent a floor effect; the absence of a statistically significant difference in PERF likely reflects insufficient sample size, as indicated by the wide confidence interval (RR 0.80, 95% CI 0.37-1.62). Whether the enhanced risk stratification and prophylactic HFNC use contribute to a true reduction in PERF requires confirmation in a larger, adequately powered study.

NPPV and HFNC as prophylactic and rescue strategies

Prophylactic NPPV is effective in preventing respiratory failure after extubation in selected high-risk patients, including those with COPD, cardiac comorbidities, advanced age, hypercapnia, or obesity [18-20].

HFNC offers several advantages over conventional oxygen therapy, including anatomical dead space washout through high flow rates, generation of a mild positive end-expiratory pressure effect that increases functional residual capacity, reduced inspiratory effort, and improved airway clearance through heated and humidified gas delivery [19,21]. These physiological effects have been shown to reduce re-intubation rates in patients at high risk for PERF [19,20,22].

In our study, the most prominent change was the increased use of prophylactic HFNC (6.2% in the EP group vs. 21.0% in the MP group; IPTW-adjusted p < 0.001). The increased use of prophylactic HFNC in the MP group may have contributed to the observed trend toward lower PERF rates, although this study was underpowered to confirm this association. HFNC was formally incorporated into Japan's National Health Insurance coverage during the April 2016 revision of medical service fees, which may partly explain its increased utilization in clinical practice. Furthermore, the MP incorporated risk stratification for re-intubation, which may also have contributed to the more frequent use of HFNC.

The effectiveness of NPPV and HFNC as rescue therapies for PERF remains limited, and early re-intubation is generally recommended [19,23]. However, NPPV has proven effective in specific conditions such as COPD exacerbation and cardiogenic pulmonary edema [19,20]. Grieco et al. reported that rescue NPPV or HFNC for PERF has a high failure rate. However, when clear criteria for re-intubation are applied, these strategies are not associated with an increase in in-hospital mortality [24]. Close monitoring and frequent reevaluation are essential to detect treatment failure promptly and avoid delayed re-intubation [19,24].

CLT and prophylactic steroid administration

The rates of failed CLT and prophylactic steroid use did not differ significantly between groups. This suggests that the assessment and management of laryngeal edema risk were appropriately implemented in both protocols. CLT is valuable for evaluating upper airway patency, and prophylactic steroid administration in patients with failed CLT has been shown to reduce the risk of re-intubation due to laryngeal edema [25,26]. Our protocol specified either methylprednisolone 20 mg administered four times starting 12 hours before extubation, or a single 40 mg dose administered four hours before extubation. Feng et al. suggested the use of methylprednisolone (20 mg every six hours) and dexamethasone (5 mg every six hours) before extubation and reported that methylprednisolone and dexamethasone are the most effective steroids for preventing post-extubation stridor and re-intubation; however, the usefulness of hydrocortisone is limited [27].

LOS and mortality

Both CCC and hospital LOS tended to be lower in the MP group, although these differences were not statistically significant.

The results of this study are comparable to those of our previous report [6], which identified an in-hospital mortality rate of 6.9%, a 30-day mortality rate of 1.2%, and a 60-day mortality rate of 4.4%. Hospital mortality was slightly higher in the MP group (4.8% vs. 8.6%, IPTW-adjusted p = 0.244), and the 28- and 60-day mortality rates were similar. A cause-of-death analysis revealed that all hospital deaths in both groups were unrelated to the extubation protocol; no deaths were attributable to airway or respiratory complications following extubation, and no deaths occurred within 48 hours of extubation (Table 6). The median time from extubation to death was 24.5 days in both groups. The higher proportion of deaths due to progression of underlying disease in the MP group (60% vs. 25%) suggests that the observed mortality difference reflects the severity of baseline conditions rather than protocol-related safety concerns. Nevertheless, the non-significant trend toward higher hospital mortality in the MP group warrants cautious interpretation and further investigation in a larger study.

Study limitations

This study had several limitations. First, it was a single-center, retrospective observational study conducted at a tertiary care facility, limiting the generalizability of the study findings to other settings. Second, the before-and-after study design, which compared two distinct periods (2017-2020 vs. 2021-2024), introduces the possibility of temporal confounding. Changes in clinical practice, staffing, equipment, and advances in critical care management over time may have influenced the outcomes independent of the protocol modifications. Third, although IPTW adjustment was used to account for measured confounders, unmeasured confounding factors could not be excluded. Fourth, the sample size may have been insufficient to detect clinically meaningful differences in outcomes, such as re-intubation rate and PERF, as suggested by trends that did not reach statistical significance. Accordingly, a larger multicenter study is needed to confirm these findings. Fifth, the MP was associated with a significantly higher use of prophylactic HFNC without a demonstrated improvement in clinical outcomes, and the cost-effectiveness and resource utilization implications of this change were not evaluated. Sixth, although the MP period was initiated after a transitional period of approximately six months, during which the nursing staff underwent training, a learning curve effect during the early phase of implementation cannot be excluded. The protocol appeared to be implemented with reasonable adherence and fidelity; nevertheless, variations in protocol compliance among clinicians and nursing staff may have attenuated the observed effects. Seventh, we excluded the transitional period (March 2020-February 2021) to ensure a clear distinction between the two protocols, as the MP was gradually introduced and medical staff underwent training during this period. Nevertheless, we cannot entirely exclude the possibility that other unmeasured temporal changes in clinical practice, including those related to the COVID-19 pandemic, influenced outcomes. Eighth, this study was not designed as a formal non-inferiority trial, and no non-inferiority margin was predefined. Therefore, the absence of statistically significant differences should not be interpreted as confirmation of equivalence. No a priori power calculation was performed, and post hoc power calculations were not conducted, as they are generally considered methodologically uninformative. Instead, the wide confidence intervals for the primary outcomes are presented as a more meaningful indicator of the limited precision in estimating treatment effects, and the findings are best regarded as hypothesis-generating rather than definitive. In this study, self-extubation was used as an exclusion criterion because these patients did not undergo protocolized extubation, consistent with our previous methodology [6]. However, in protocols incorporating nurse-led daily SATs, self-extubation events may be related to the intervention itself, and future studies should include these events as a protocol-related outcome rather than an exclusion criterion. Finally, the long-term outcomes beyond hospital discharge, such as functional status and quality of life, were not assessed.

Future directions

Future studies should consider multicenter collaborative designs to establish external validity and prospective randomized controlled trials to establish causality and optimize weaning strategies based on patient-specific risk stratification. Further research should focus on developing artificial intelligence-based weaning prediction models and evaluating long-term outcomes, including quality of life and functional status.

## Conclusions

In this exploratory before-and-after study, the implementation of a modified extubation protocol incorporating nurse-led daily SATs and systematic risk assessment was not associated with a statistically significant increase in re-intubation rates or PERF. The MP was, however, associated with a significantly higher use of prophylactic HFNC. Given the limited statistical power and absence of a predefined non-inferiority margin, these findings should be considered hypothesis-generating. A larger multicenter study with an appropriate non-inferiority design is warranted to confirm the safety and efficacy of this protocol.

## References

[1] Epstein SK, Ciubotaru RL, Wong JB (1997). Effect of failed extubation on the outcome of mechanical ventilation. Chest.

[2] Thille AW, Harrois A, Schortgen F, Brun-Buisson C, Brochard L (2011). Outcomes of extubation failure in medical intensive care unit patients. Crit Care Med.

[3] Menon N, Joffe AM, Deem S (2012). Occurrence and complications of tracheal reintubation in critically ill adults. Respir Care.

[4] Ely EW, Baker AM, Dunagan DP (1996). Effect on the duration of mechanical ventilation of identifying patients capable of breathing spontaneously. N Engl J Med.

[5] Girard TD, Kress JP, Fuchs BD (2008). Efficacy and safety of a paired sedation and ventilator weaning protocol for mechanically ventilated patients in intensive care (awakening and breathing controlled trial): a randomised controlled trial. Lancet.

[6] Nitta K, Okamoto K, Imamura H (2019). A comprehensive protocol for ventilator weaning and extubation: a prospective observational study. J Intensive Care.

[7] Committee for the Liberation From Mechanical Ventilation Guidelines, Japanese Society of Intensive Care Medicine (2015). Liberation from mechanical ventilation guidelines 2015. J Jpn Soc Intensive Care Med.

[8] Kikuchi T, Toba S, Sekiguchi Y (2011). Protocol-based noninvasive positive pressure ventilation for acute respiratory failure. J Anesth.

[9] Stollings JL, Devlin JW, Lin JC, Pun BT, Byrum D, Barr J (2020). Best practices for conducting interprofessional team rounds to facilitate performance of the ICU liberation (ABCDEF) bundle. Crit Care Med.

[10] Knaus WA, Draper EA, Wagner DP, Zimmerman JE (1985). APACHE II: a severity of disease classification system. Crit Care Med.

[11] Kanda Y (2013). Investigation of the freely available easy-to-use software 'EZR' for medical statistics. Bone Marrow Transplant.

[12] Kress JP, Pohlman AS, O'Connor MF, Hall JB (2000). Daily interruption of sedative infusions in critically ill patients undergoing mechanical ventilation. N Engl J Med.

[13] Wang Y, Wang Y, Gu S, Zhu L, Jia R, Tan M, Ma S (2025). Nurse-led weaning protocols-a systematic review and meta-analysis. Front Med (Lausanne).

[14] Ketcham SW, Adie SK, Brummel K, Walker E, Prescott HC, Thomas MP (2022). Implementation of a nurse-driven spontaneous awakening trial protocol in a cardiac intensive care unit. Crit Care Nurse.

[15] Alkhateeb T, Semler MW, Girard TD, Ely EW, Stollings JL (2025). Comparison of SAT and SBT conduct during the ABC trial and PILOT trial. J Intensive Care Med.

[16] Miller MA, Krein SL, George CT, Watson SR, Hyzy RC, Iwashyna TJ (2013). Diverse attitudes to and understandings of spontaneous awakening trials: results from a statewide quality improvement collaborative. Crit Care Med.

[17] Olsen GH, Gee PM, Wolfe D (2023). Awakening and breathing coordination: a mixed-methods analysis of determinants of implementation. Ann Am Thorac Soc.

[18] Ouellette DR, Patel S, Girard TD (2017). Liberation from mechanical ventilation in critically ill adults: an official American College of Chest Physicians/American Thoracic Society clinical practice guideline: inspiratory pressure augmentation during spontaneous breathing trials, protocols minimizing sedation, and non-invasive ventilation immediately after extubation. Chest.

[19] Munshi L, Mancebo J, Brochard LJ (2022). Noninvasive respiratory support for adults with acute respiratory failure. N Engl J Med.

[20] Fernando SM, Tran A, Sadeghirad B (2022). Noninvasive respiratory support following extubation in critically ill adults: a systematic review and network meta-analysis. Intensive Care Med.

[21] Nishimura M (2016). High-flow nasal cannula oxygen therapy in adults: physiological benefits, indication, clinical benefits, and adverse effects. Respir Care.

[22] Hernández G, Vaquero C, González P (2016). Effect of postextubation high-flow nasal cannula vs conventional oxygen therapy on reintubation in low-risk patients: a randomized clinical trial. JAMA.

[23] Esteban A, Frutos-Vivar F, Ferguson ND (2004). Noninvasive positive-pressure ventilation for respiratory failure after extubation. N Engl J Med.

[24] Grieco DL, Jaber S, Zakynthinos S (2025). Use of rescue noninvasive ventilation for post-extubation respiratory failure. Crit Care.

[25] Kuriyama A, Umakoshi N, Sun R (2017). Prophylactic corticosteroids for prevention of postextubation stridor and reintubation in adults: a systematic review and meta-analysis. Chest.

[26] Girard TD, Alhazzani W, Kress JP (2017). An official American Thoracic Society/American College of Chest Physicians clinical practice guideline: liberation from mechanical ventilation in critically ill adults. Rehabilitation protocols, ventilator liberation protocols, and cuff leak tests. Am J Respir Crit Care Med.

[27] Feng IJ, Lin JW, Lai CC (2023). Comparative efficacies of various corticosteroids for preventing postextubation stridor and reintubation: a systematic review and network meta-analysis. Front Med (Lausanne).

